# Immunological characteristics of Spanish IDUs infected with HTLV-2 and HIV-1

**DOI:** 10.1186/1742-4690-8-S1-A96

**Published:** 2011-06-06

**Authors:** Maria Abad, Fernando Dronda, Ana Moreno, Maria J Perez-Elias, Ester Dominguez, Santiago Moreno, Alejandro Vallejo

**Affiliations:** 1 Department of Infectious Diseases, Hospital Ramon y Cajal, Madrid, 28035, Spain

## Background

HTLV-2 infection has been detected in injecting drug users (IDUs) in Spain, who often are coinfected with HCV and HIV-1. It has been reported that HCV infection increases the immune activation of HIV-1-infected patients. Our objective was to analyze how HTLV-2 infection affects T-cell activation and other immunological parameters such as naïve and memory T-cells and immunosenescence.

## Methods

Eighteen HTLV-2 positive IDUs from Spain, also infected with HCV and HIV-1 were selected for this study. Fresh blood was immunostained with CD38+/HLA-DR+ antibodies to define activated cells, CD45RA+/CD62L+ for naïve T-cells, CD45RA-/CD62L- for effector memory T-cells and CD57+ for T-cell senescence, in both CD4+ and CD8+ cells. Immunological parameters were compared to two control groups, HCV-HIV-1 coinfected (N=7) and HIV-1 monoinfected patients (N=7).

## Results

The median of CD4+ and CD8+ counts for the HTLV-2-infected individuals were 497[312 632] and 784[598-1206], respectively. CD4+ count was lower than control groups, with no statistical difference. Among CD4+ cells, naïve cells decreased while effector memory cells increased. No significant differences were found in CD8+ level and senescence when compared to group controls. CD8+ cell activation (8.5[2.93-12.21] was higher than control groups, either HCV-HIV-1-coinfected patients (4.02[3.54-6.11], p=0.038) or HIV-1-monoinfected patients (5.7[2.72-8.32], p=0.056).

## Conclusions

HTLV-2 induces an increase of CD8+ cell activation and senescence in HCV-HIV-1 coinfected patients compared to HCV-HIV-1 or HIV-1 infection. Besides, the levels of CD4+ count and CD4+ naïve cells are lower in these patients. Nevertheless, the CD4+ effector memory cells increased. This might have beneficial effects on HIV-1 or HCV infection.

